# Dual KS: Defining Gene Sets with Tissue Set Enrichment Analysis

**DOI:** 10.4137/cin.s2892

**Published:** 2010-01-21

**Authors:** Yarong Yang, Eric J. Kort, Nader Ebrahimi, Zhongfa Zhang, Bin T. Teh

**Affiliations:** 1 Laboratory of Molecular Epidemiology, Van Andel Research Institute, Grand Rapids, MI; 2 Laboratory of Cancer Genetics, Van Andel Research Institute, Grand Rapids, MI; 3 These authors contributed equally to this work; 4 Division of Statistics, Northern Illinois University, De Kalb, IL

**Keywords:** gene set enrichment analysis (GSEA), gene expression, DKS algorithm, gene signatures

## Abstract

**Background:**

Gene set enrichment analysis (GSEA) is an analytic approach which simultaneously reduces the dimensionality of microarray data and enables ready inference of the biological meaning of observed gene expression patterns. Here we invert the GSEA process to identify class-specific gene signatures. Because our approach uses the Kolmogorov-Smirnov approach both to define class specific signatures and to classify samples using those signatures, we have termed this methodology “Dual-KS” (DKS).

**Results:**

The optimum gene signature identified by the DKS algorithm was smaller than other methods to which it was compared in 5 out of 10 datasets. The estimated error rate of DKS using the optimum gene signature was smaller than the estimated error rate of the random forest method in 4 out of the 10 datasets, and was equivalent in two additional datasets. DKS performance relative to other benchmarked algorithms was similar to its performance relative to random forests.

**Conclusions:**

DKS is an efficient analytic methodology that can identify highly parsimonious gene signatures useful for classification in the context of microarray studies. The algorithm is available as the dualKS package for R as part of the bioconductor project.

## Background

Gene set enrichment analysis (GSEA), first described by Subramanian et al,^1^ is an analytic approach which simultaneously reduces the dimensionality of microarray data and enables ready inference of the biological meaning of observed gene expression patterns. GSEA entails grouping genes together into either empirically or theoretically defined signatures. Combining genes improves the signal to noise ratio of the data since the random variation in multiple genes tend to cancel each other out. This decrease in variability favors smaller sample sizes and more efficient study design. Furthermore, to the extent that the signatures are defined in some systematic fashion, GSEA facilitates inference as to the biological processes that are relevant to the phenotype being studied. This approach has been used by ourselves and others to identify novel biologic characteristics of tumor samples.^2^–^5^

Here we extend this analytic methodology to the task of tissue diagnosis. We invert the GSEA process described above to identify class-specific gene signatures that may subsequently be used for sample classification. Rather than looking for bias of a subset of genes in an ordered list of expression levels for a given sample, we look for bias of a subset of samples in an ordered list of samples for a given gene. By applying this methodology across the genome, we can identify those genes whose expression is most strongly biased in a given subset of samples. This can be conceptualized as “tissue-set enrichment analysis”.

The approach to quantifying gene set enrichment described by Subramanian et al utilizes a variation of the Kolmogorov Smirnov rank-sum statistic (KS). KS measures how biased a subset of items is in the ordered list of all items.^1^,^6^ In the context of GSEA, genes are sorted based on expression level for a given sample, and then KS measures the extent to which the genes in a given signature occur early or late in that ordered list as opposed to being randomly distributed throughout the list. We use tumor subtype to define the subsets and the result is a gene signature defining a specific tumor class. These signatures can then be applied to classification of unknown samples using the traditional GSEA approach. Since the GSEA approach measures the extent to which a set of genes is highly expressed in a given sample *relative to all genes*, it is important that the selected genes satisfy both of the following requirements:

1. The selected genes are specific: they are over-(or under-) expressed in the class of interest relative to other classes and
2. The selected genes are distinctive: The selected genes have the highest (or lowest) expression in samples of the class of interest among all over- (or under-) expressed genes.

Because our approach uses the KS approach both to define class specific signatures and to classify samples using those signatures, we have termed this methodology “Dual-KS” (DKS). Here we compare our algorithm to alternative classification methods and also examine the effects of several variations to the algorithm. Software implementing the algorithm is available as the dualKS package through the bioconductor project (http://www.bioconductor.org).

The approach has several advantages. First, it is well suited to identifying signatures amenable to use for GSEA-style classification. Second, it is non-iterative and determinant; i.e. it is computationally efficient and produces exactly the same gene set from run to run. Third, it produces highly parsimonious gene signatures amenable to downstream validation. Small gene sets are desirable for focusing subsequent laboratory investigation, or when clinical assay development is contemplated using low- or medium-throughput assay technologies. Finally, the algorithm is well suited to identifying gene signatures that are unique to a particular class of samples even where more than two classes are considered-the “multi-class case”-as is the case, for example, in renal tumors for which there are many histological subtypes to be distinguished.

Among alternative approaches to discriminant analysis and classification, we would like to highlight the gene selection methodology described by Diaz-Uriarte and Alvarez de Andres,^7^ which is an adaptation of the random forest algorithm first described by Breiman.^8^ This approach also is designed to identify highly parsimonious gene signatures, including unique gene signatures in the multi-class case. They have previously benchmarked their algorithm against several common alternatives, namely support vector machines (SVM),^9^ K-nearest neighbors (KNN) with and without variable selection,^10^ diagonal linear discrimination analysis (DLDA),^10^ and nearest shrunken centroids (SC).^11^ We are indebted to their thorough treatment of these alternative methodologies and we refer here to their benchmarking results for comparison.

There are a variety of other alternatives that could be considered. A more basic approach to discriminant analysis is to use statistical tests such as t-tests or wilcoxon rank sum tests to compare expression levels of each gene among two groups of samples. However, in the multi-class case, it is not always obvious which pairwise comparisons are biologically most meaningful. For example, if classes A, B, and C are to be compared, should the comparisons be A vs. C and B vs. C, or A vs. B+C and B vs. A+C? And if the former type of comparison is made, the identified gene signatures may be not be unique since the same gene may distinguish both A from C and B from C. More sophisticated methodologies such as biclustering^12^ are well suited to the multi-class case, but may likewise identify genes characteristic of several (though not all) of the possible classes. The COPA algorithm, initially developed to identify candidate chromosomal translocations, 13 can identify gene pairs that are deregulated relative to normal or other reference samples in mutually exclusive subsets of disease related samples. However, these subsets have traditionally been data driven, i.e. identified based on expression levels of the gene pair in question and not based on phenotype as identified by the investigator. Presumably the approach could be adapted to sample classes fixed a *priori* by the investigator, in which case the approach could identify discriminant gene pairs for up to two classes of samples relative to reference samples. Finally, the PPST algorithm^14^ is based on quantile scores of gene expression values in normal and disease tissues. While designed for the two class case, this algorithm has the unique feature of identifying genes that are very high in a subset of the disease samples relative to normal, but very low in a separate subset. While we do not benchmark DKS against these algorithms (because they do not identify unique gene signatures, are not designed to address the multi-class case, and/or do not describe an unique, analogous methodology for classification of new samples after gene selection), each has important strengths and represent useful alternatives in appropriate experimental contexts.

In the balance of this paper we describe in detail our algorithm and its variants. We then estimate its error rate and compare it to the previously published methods mentioned above. As will become apparent, no single methodology is suitable in every circumstance. However, our DKS algorithm can efficiently produce extremely small yet highly robust gene signatures in many situations and therefore we propose that it is worthy of consideration for inclusion in the gene expression analysis workflow.

## Results and Discussion

### Algorithm

#### Identification of discriminant genes

Given a *G* × *N* gene expression matrix *X* for *G* genes and *N* samples and a classification vector *Y* = (*y*_1_, …, *y_N_*), where *y*_j_ is the biological classification for sample *j*, we calculate the matrix *U*, as follows. For each gene *i*, we sort its *N* expression values in decreasing order to identify the degree of upregulation of each gene in each class. For each of the *N* samples ordered from the highest to lowest based on their expression values in row *i* we let

$$a_{ilj}=\{\begin{array}{c} \frac{N}{N_{l}},\;\;\;\text{if}\;\text{sample}\;j\;\text{is}\;\text{of}\;\text{class}\;l\\ -\frac{N}{N-N_{l}},\;\;\;\text{otherwise} \end{array}$$

where *j* is the index of the ordered list of the *N* expression values for gene *i*, and *l* is the class among *K* unique classes in *Y. N_l_* denotes the number of samples of class *l* in the complete set of *N* samples. We then define the scoring function

(1) $$u_{il}=\text{max}\sum_{j=1}^{N}a_{ilj}.$$

The result is a *G* × *K* matrix *U*, such that for each gene we have the maximum of the running sum for each class. By sorting the genes based on decreasing *u_il_* for a given class, we can identify those genes that are most upwardly biased in a specific class in terms of their ordered expression levels.

On the other hand, for each gene *i*, we sort its *N* expression values in increasing order to identify the degree of downregulation of each gene in each class. For each of the *N* samples ordered from lowest to highest based on their expression values in row *i* we let

$$b_{ilj}=\{\begin{array}{c} \frac{N}{N_{l}},\;\;\;\text{if}\;\text{sample}\;j\;\text{is}\;\text{of}\;\text{class}\;l\\ -\frac{N}{N-N_{l}},\;\;\;\text{otherwise} \end{array}$$

We then compute the scoring function

(2) $$d_{il}=\text{max}\sum_{j=1}^{N}b_{ilj}.$$

Actually, it is not hard to recognize that

$$d_{il}=-\text{min}\sum_{j=1}^{N}a_{ilj}$$

in the case that the *N* expression values are sorted in decreasing order. The result is a *G* × *K* matrix *D*, such that for each gene we have the maximum of the running sum for each class for the genes sorted in increasing order. By sorting the genes based on decreasing *d_il_* for a given class, we can identify those genes that are most downwardly biased in a specific class in terms of their ordered expression levels.

To illustrate the computation of the scoring functions (1) and (2), we let *N* = 8 and *K* = 3. Suppose, for gene *i*, *i* = 1, the 8 expression values are 1088.3, 841.9, 762.8, 681.2, 744.0, 878.7, 660.1, and 1163.2. These 8 samples correspond to classes C1, C1, C2, C3, C1, C2, C3, and C2, respectively. So *N*_1_ = 3, *N*_2_ = 3, and *N*_3_ = 2. By sorting these 8 values in decreasing order we have 1163.2, 1088.3, 878.7, 841.9, 762.8, 744.0, 681.2, and 660.1 corresponding to classes C2, C1, C2, C1, C2, C1, C3, and C3, respectively. To compute *u*_11_ which denotes the degree of upregulation of gene 1 in class C1, we calculate *a*_11_*_j_*, *j* = 1, …, 8. They are −8/5, 8/3, −8/5, 8/3, −8/5, 8/3, −8/5, and −8/5. Based on function (1), for *j* = 1, …, 8, we have the running sum as −8/5, 16/15, −8/15, 32/15, 8/15, 16/5, 8/5, and 0. Therefore, *u*_11_ is 16/5 which is the maximum of the running sum. Similarly, we can compute *u*_12_ and *u*_13_ which denote the degree of upregulation of gene 1 in class C2 and C3 respectively. One can repeat this process for all the *G* genes to get the matrix *U*.

To compute *d*_11_, the gene expression values are sorted as 660.1, 681.2, 744.0, 762.8, 841.9, 878.7, 1088.3, and 1163.2 corresponding to class C3, C3, C1, C2, C1, C2, C1, and C2, respectively. *b*_11_*_j_*, *j* = 1, …, 8 are computed. They are −8/5, −8/5, 8/3, −8/5, 8/3, −8/5, 8/3, and −8/5. Based on function (2), for *j* = 1, …, 8, we have the running sum as −8/5, −16/5, −8/15, −32/15, 8/15, −16/15, 8/5, and 0. Therefore, *d*_11_ is 8/5. By repeating this process for all the *G* genes we can get the matrix *D*.

One limitation of this approach is that for a given gene, some samples of a given class may occur very early in the ordered list (leading to an elevated value for *u*), while other samples of that class occur verylate in the list (leading to an elevated value for *d*). In the worst case scenario, half may occur at the very beginning of the list, and half at the very end. This is arguably the worst possible classifier. To penalize such genes, we can take as our final score the difference between *u* and *d*. Therefore, we denote the matrix *U*^Δ^ = *U* – *D* = (*u_il_* – *d_il_*), and for each class *l* select genes with the highest scores in column *l* of the matrix *U*^Δ^. These genes are selected because their expression in class *l* is higher than in other classes.

Likewise, we denote the matrix *U*^Δ^ = *D* – *U* = (*d_il_* – *u_il_*) and for each class *l* select genes with highest scores in the column l of this matrix. To guarantee equal weight to each class in the classification stage which will be described later, we pick equal number of genes for each class. We identify the optimum number of genes per class through cross validation.

#### Variation 1: Weighted dualKS score

Another potential pitfall of our approach may occur when a gene has high expression in a given class relative to other classes, but not relative to other genes. Since we will subsequently calculate KS statistics gene-wise (rather than sample-wise) in the classification step, it is important that the selected genes not only have biased expression in the class of interest, but also be among the highest (or lowest) expressed genes. To favor genes that satisfy both requirements, as opposed to only the first requirement, we can weight each gene according to its average expression in a particular class. We defined the weight for gene *i* and class *l* as:

$$w_{il}=-\text{log}\frac{{\bar{R}}_{il}}{G},$$

where *G* is the total number of genes and *R̄_il_* is the *rank* of gene *i*’s average expression in class *l* among the *G* genes’ average expression values in class *l*. As an illustration, suppose we have the following average expression matrix with *G* = 3 and *K* = 3,

$$[\begin{array}{cccc} & \text{C}1 & \text{C}2 & \text{C}3\\ g1 & 823.64 & 777.64 & 652.38\\ g2 & 987.77 & 486.87 & 878.98\\ g3 & 678.98 & 123.68 & 268.98 \end{array}].$$

Here, for example for the gene *i* = 1 and the class *l* = 1, *R̄*_11_ is 2 because in the first column the rank of 823.64 is 2. Consequently, we have *w*_11_ = − log2/3. Bases on this weighting strategy, genes with highest absolute expression in a given class are more likely to be included in the signature for that class. The weighted scoring functions *û* and *d̂* are then as follows:

$${\hat{u}}_{il}=w_{il}u_{il}$$

and

$${\hat{d}}_{il}=(1-w_{il})d_{il}.$$

And the corresponding weighted score matrices are denoted *Û*^Δ^ and *D̂*^Δ^.

#### Variation 2: Rescaled dualKS score

As an alternative to weighting, we also investigated the utility of rescaling the calculated KS statistic by an empirically determined scaling factor such that the range of possible scores is constrained to fall between 0 and 1. The scaling factor is simply the maximum score obtained for a given signature among the training samples. When the KS statistic is divided by this scaling factor, arbitrary differences in the expression level between signatures is eliminated.

#### Classification

Once a suitable gene signature has been described, new samples may be classified based on their expression values *x* = (*x*_1_, *x*_2_,…, *x_n_*; *x_n_*_+1_, *x_n_*_+2_,…, *x*_2_*_n_*), where (*x*_1_, *x*_2_, …, *x_n_*) and (*x_n_*_+1_, *x_n_*_+2_, …, *x*_2_*_n_*) denote the expression values of upregulated gene signature and downregulated gene signature respectively. Here, the enrichment score of each class-specific signature is determined, and the sample is assigned to the class whose signature achieves the highest score for that sample. Specifically, for each class *l* we define the signature for that class as the t highest scoring genes from $U_{.l}^{\Delta }$ and $D_{.l}^{\Delta }$ (or, for the weighted case, ${\hat{U}}_{.l}^{\Delta }$ and ${\hat{D}}_{.l}^{\Delta }$), denoted u_l_^*^ and d_l_^*^ for the up and down regulated genes, respectively. As stated above, to guarantee equal weight to each class, we pick equal number of genes for each class. Therefore, there exists *n* = *t* × *K*, where *t* is determined empirically.

We then sort the *n* upregulated gene expression values (*x*_1_, *x*_2_, …, *x_n_*) in decreasing order and let

$$a_{il}^{\prime }=\{\begin{array}{c} \frac{n}{n_{l}},\;\;\;\text{if}\;\text{gene}\;i\in u_{l}^{*}\\ -\frac{n}{n-n_{l}},\;\;\;\;\text{otherwise} \end{array}$$

where *n_l_* is the number of upregulated genes in the signature of class *l*. We then sort the *n* downregulated gene expression values (*x_n_*_+1_, *x_n_*_+2_, …, *x*_2_*_n_*) in increasing order and let

$$b_{il}^{\prime }=\{\begin{array}{c} \frac{n}{n_{l}},\;\;\;\text{if}\;\text{gene}\;i\in d_{l}^{*}\\ -\frac{n}{n-n_{l}},\;\;\;\;\text{otherwise} \end{array}$$

where *n_l_* is the number of downregulated genes in the signature of class *l*. We compute two scoring functions

$$u_{l}^{\prime }=\text{max}\sum_{i=1}^{n}a_{il}^{\prime }$$

and

$$d_{l}^{\prime }=\text{max}\sum_{i=n+1}^{2n}b_{il}^{\prime }.$$

The enrichment score *E_l_* is then calculated as the sum of the scores for the upregulated and downregulated gene signatures for class *l*:

$$E_{l}=u_{l}^{\prime }+d_{l}^{\prime }$$

or, for the rescaled case:

$$E_{l}=\frac{u_{l}^{\prime }+d_{l}^{\prime }}{r_{l}}$$

where *r_l_* is the rescaling factor for that class. The sample is assigned to the class corresponding to the maximum *E_l_*.

Note that classifiers are not necessary to be composed of both of the upregulated and downregulated gene signatures. For example, if only upregulated gene signature is used, then $E_{l}=u_{l}^{\prime }$ or $E_{l}=u_{l}^{\prime }/r_{l}$ rescaled case.

#### Testing

**Figure 1:**
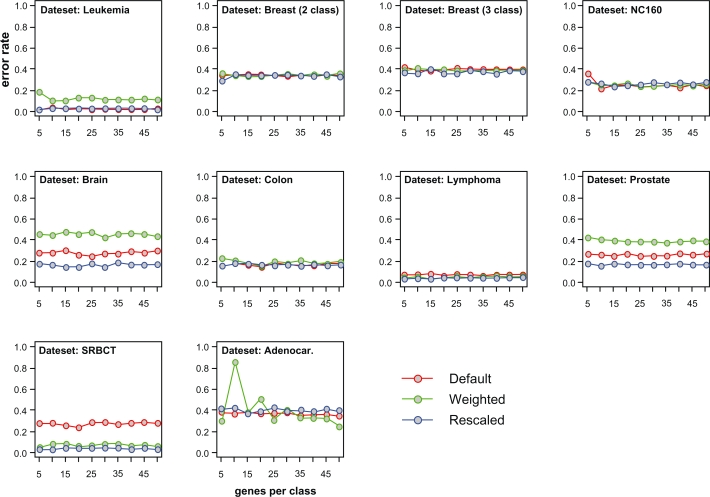
**Comparison of DKS variants.** For each of the 10 test datasets, the error rate is plotted as a function of upregulated gene signature size (number of genes per class) ranging from 5 to 50 with an increment of 5. The three variations (default, weighted KS score, and rescaled KS score) are plotted to allow comparison of these methodologies.

In order to test our algorithm, we downloaded published data^6,15–22^ that have been used in a previous study benchmarking classification methodologies.^7^ We used the same error rate estimation algorithm that was used in that paper (0.632+ bootstrap),^23^ allowing direct comparison of our algorithm to the algorithms tested previously. Error rates were estimated for each of the 10 benchmarking datasets using the default, weighted, and rescaled versions of our dualKS algorithm, and using upregulated gene signatures ranging between 5 and 50 (with an increment of 5) genes per class. Note that the range 5–50 and the increment of 5 were selected for illustration purpose. In fact, the range can start from 1 and end at a number different from 50 and the best increment should be 1 because we use the optimum gene signature to estimate the error rate. The reason we use the range 5–50 and the increment of 5 in this paper is to make the comparison of DKS variants (Fig. 1) clearer. With our selected range and increment, it is shown that for most of the datasets, increasing the size of the gene signature above 15 genes per class did not result in an improvement in error rates and in some cases (Fig. 1) larger signatures performed more poorly than smaller ones, suggesting overfitting. The three variations of the DKS algorithm were roughly equivalent in terms of error rates for 6 of the 10 datasets. Where differences were observed, the rescaled variant always performed well, with the default algorithm performing more poorly in three of the 10 datasets, and the weighted algorithm performing more poorly in three datasets as well.

The estimated error rate of DKS using the optimum gene signature was smaller than the estimated error rate of random forest in 4 out of the 10 datasets, and was equivalent in two additional datasets (Table 1). DKS outperformed the other benchmarked algorithms to a similar degree.

The optimum gene signature size for each dataset was identified as the gene set size that achieved the lowest estimated error rate (Table 2). One advantage of the random forest algorithm is that it favors parsimonious gene signatures. Nevertheless, the optimum gene signature identified by the DKS algorithm was smaller than the random forest gene signature in 5 out of 10 datasets.

### Implementation, availability and requirements

**Figure 2:**
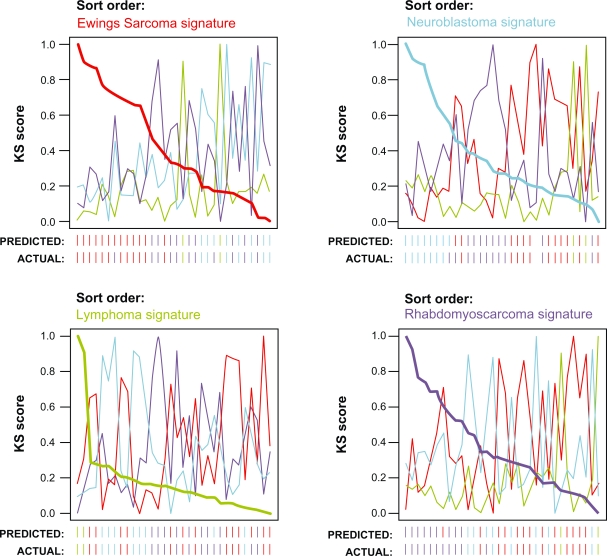
**Sample output of dualKS package.** Shown is a plot of analysis of the SRBCT dataset generated by the dualKS package implementing the DKS algorithm. The data is plotted sorted by each class signature in turn so that the relationship between high and low scoring samples on each class may be inspected. The actual and predicted classes of each sample are indicated below the X axis.

The DKS algorithm has been implemented in the dualKS package for *R*. The package performs both training (gene signature identification) and classification. These two steps are separated such that the identified gene signatures can be exported for use in other applications, and signatures identified by other methodologies can be utilized in the KS-based classification implemented in the package. The package also defines a custom plot function to generate summary classification plots as shown in Figure 2.

The dualKS package is freely available through the bioconductor project (http://www.bioconductor.org/packages/2.3/bioc/html/dualKS.html). The package is open source and can run on any operating system that is capable of running *R*.

## Conclusions

The DKS algorithm performs discriminant analysis based on tissue enrichment analogous to gene set enrichment used for classification. As such, it is a natural and intuitive choice for defining class-specific gene sets to be used in subsequent GSEA-type analyses. Furthermore, DKS can efficiently identify highly parsimonious gene signatures amenable to downstream validation. While no algorithm demonstrates superior performance in every context, DKS is an attractive methodology worthy of consideration for inclusion in microarray analysis workflow

## Authors Contributions

EJK conceived of the project. YY and EJK developed the algorithms, wrote the software, performed the analyses, and drafted the manuscript. NE and ZZ assisted with analysis. NE and BTT provided support and guidance for the project and reviewed the manuscript.

## Figures and Tables

**Table 1:** **Estimated error rates of various classification methods.**Comparison of error rates estimated by the 0.632+ bootstrap method. Error rates were estimated for the dualKS method (rescaled variant) and compared to previously published estimates for the other methods, reproduced in the table.

**Data set**	**Classes**	**SVM**	**KNN**	**DLDA**	**SC.l**	**SC.s**	**NN.vs**	**RF**	**DKS**
Leukemia	2	1.4	2.9	2.0	2.5	6.2	5.6	5.1	2.2
Breast (2 cl.)	2	32.5	33.7	33.1	32.4	32.6	33.7	34.2	29.1
Breast (3 cl.)	3	38.0	44.9	37.0	39.6	40.1	42.4	35.1	35.7
NCI 60	8	25.6	31.7	28.6	25.6	24.6	23.7	25.2	21.9
Adenocar.	2	20.3	17.4	19.4	17.7	17.9	18.1	12.5	23.9
Brain	5	13.8	17.4	18.3	16.3	15.9	19.4	15.4	14.1
Colon	2	14.7	15.2	13.7	12.3	12.2	15.8	12.7	14.9
Lymphoma	3	1.0	0.8	2.1	2.8	3.3	4.0	0.9	3.4
Prostate	2	6.4	10.0	14.9	8.8	8.9	8.1	7.7	15.8
SRBCT	4	1.7	2.3	1.1	1.2	2.5	3.1	2.1	2.2

**Table 2:** **Gene set sizes.**Comparison of size of gene signature identified by random forest method and DKS (rescaled variant). Since DKS identifies an independent signature for each class, both the genes per class and the total number of genes across all classes are listed.

**Data set**	**Random Forest**	**DKS (genes per class)**	**DKS (total genes)**
Leukemia	2	5	10
Breast (2 cl.)	14	5	10
Breast (3 cl.)	110	10	30
NCI 60	230	10	80
Adenocar.	6	50	100
Brain	22	15	75
Colon	14	20	40
Lymphoma	73	15	45
Prostate	18	10	20
SRBCT	101	5	20
